# The mildly decreased preoperative bilirubin level is a risk factor for periprosthetic joint infection after total hip and knee arthroplasty

**DOI:** 10.1186/s42836-021-00096-2

**Published:** 2021-12-01

**Authors:** Jun Fu, Xiyue Chen, Ming Ni, Xiang Li, Libo Hao, Guoqiang Zhang, Jiying Chen

**Affiliations:** 1 Senior Department of Orthopedics, The Fourth Medical Center of Chinese PLA General Hospital, Beijing, China; 2 National Clinical Research Center for Orthopedics, Sports Medicine & Rehabilitation, Beijing, China; 3 Department of Orthopedics, The First Medical Center of Chinese PLA General Hospital, Beijing, China; 4 Department of Orthopaedics, Sanya People’s Hospital, Sanya, 572000 China

**Keywords:** Arthroplasty, Periprosthetic joint infection, Bilirubin, Risk factor

## Abstract

**Background:**

Many serologic markers are routinely tested prior to joint arthroplasty, but only few are commonly used to guide surgeons in determining patients most at risk of periprosthetic joint infection (PJI). The objective of this study was to investigate the association between preoperative bilirubin level and PJI after primary hip and knee arthroplasty.

**Methods:**

A retrospective analysis was performed on patients undergoing revision hip and knee arthroplasty at our hospital from January 2016 to December 2019. Laboratory biomarkers were collected before the primary arthroplasty, as well as general patient information. The association between the above serologic markers and postoperative PJI was analyzed.

**Results:**

A total of 72 patients (30 hips/42 knees) were analyzed, including 39 patients with PJI and 33 patients without PJI. Except for total bilirubin (TB) and direct bilirubin (DB), there was no significant difference between the remaining laboratory biomarkers. The preoperative TB and DB in the PJI group were 10.84 ± 0.61 μmol/L and 3.07 ± 0.19 μmol/L, respectively, which were lower than those in the non-PJI group (14.68 ± 0.75 μmol/L and 4.70 ± 0.39 μmol/L, *P* < 0.001). The area under the curve (AUC) of preoperative TB to predict PJI was 0.755 (*P* < 0.001, cutoff = 11.55 μmol/L, sensitivity = 66.67%, specificity = 75.76%). Meanwhile, the AUC of preoperative DB was 0.760 (*P* < 0.001, cutoff = 4.00 μmol/L, sensitivity = 84.62%, specificity = 54.45%).

**Conclusions:**

The serum levels of TB and DB before the primary arthroplasty were lower in PJI patients than in non-PJI patients, and the preoperative values lower than 11.55 μmol/L and 4.00 μmol/L could be considered as a risk factor for postoperative PJI.

## Introduction

Periprosthetic joint infection (PJI) is one of the catastrophic complications following joint arthroplasty that actually increases financial burden and suffering to the patients and their families [1]. PJI is the number one cause of failure in total knee arthroplasty (TKA) and the third leading cause of failure in total hip arthroplasty (THA) [2, 3]. The reported incidence of PJI is 1–3% following primary arthroplasty and 3–5% after revision arthroplasty [4, 5]. With prolonged life expectancy and a growing indication for primary joint arthroplasty, there will be a fold increase in the number of PJI patients [6]. Early and accurate identification of individuals at high risk of PJI is conductive to clinical decision-making and development of effectively preventive strategies.

Given the severity of PJI, previous studies have identified a tremendous number of risk factors for PJI [7, 8]; they can be divided into the patient-related (intrinsic factor) and environment-related (extrinsic factor) factors and play a crucial part in pre-, intra- or postoperative periods. Many serologic markers are routinely tested before joint arthroplasty, but only few are commonly used to guide surgeons in determining patients most at risk of PJI [9]. Among these biomarkers are C-reactive protein (CRP), erythrocyte sedimentation rate (ESR), D-dimer, fibrinogen, and other visceral organ specific biomarkers, and they are mainly used to monitor or detect comorbidities, such as diabetes, inflammatory arthritis, renal disease, immunosuppression, and malnutrition, among others. The values may be affected by the pre-existing comorbidities and misguide the surgeons’ diagnosis for PJI.

Recently, it has been reported that mildly elevated bilirubin levels in adults were protective against pathologies such as diabetes type 2, cardiovascular diseases, and several cancers, supposedly due to its powerful anti-inflammatory and anti-oxidative effect [10, 11]. Previous studies have found that bilirubin impairs bactericidal activity of neutrophils through scavenging reactive oxygen species (ROS) and increasing NADPH oxidase-1 (NOX-1) and cyclooxygenase-2 (COX-2) in patients with hyperbilirubinemia, resulting in physiologic effects mitigated by increased antioxidant activity [12, 13].

Laky B et al [14] reported that mildly decreased preoperative bilirubin levels with a cutoff at 8.72 μmol/L or 0.51 mg/dL were significantly associated to patients with PJI after shoulder and knee arthroplasty. To our knowledge, there are no other studies on the correlation between serum bilirubin levels and PJI. Therefore, we conducted a retrospective case-control and large sample study to compare preoperative serum bilirubin levels between patients with and without PJI after hip and knee arthroplasties and to confirm the hypothesis that patients with PJI, compared to without PJI after THA and TKA, would present with lower preoperative bilirubin levels.

## Materials and methods

### Patients

**Fig. 1:**
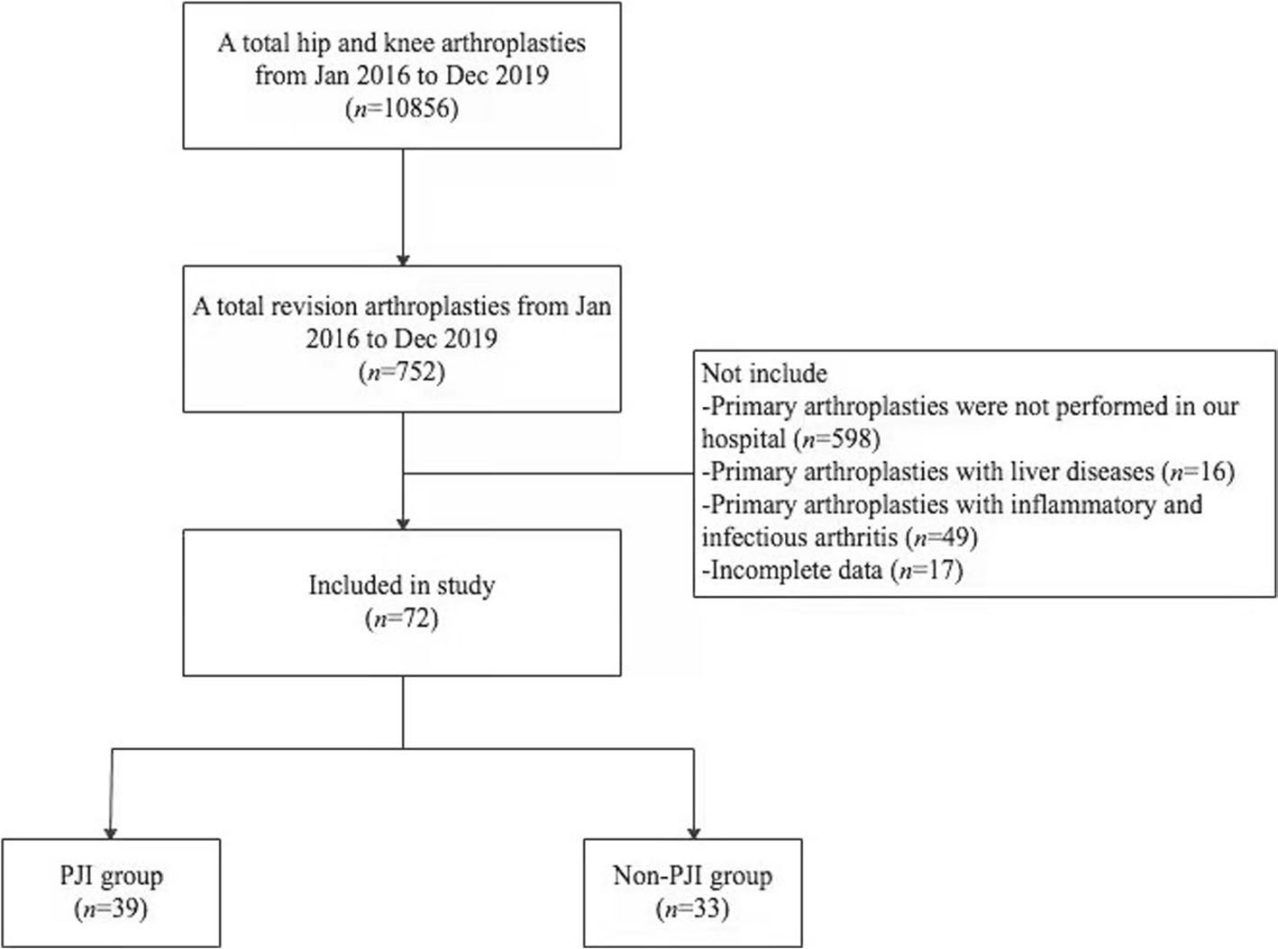
Patient inclusion and exclusion flowchart

After receiving approval from the institutional review board at our hospital, a retrospective analysis was performed on patients undergoing revision hip and knee arthroplasty at our center from January 2016 to December 2019 (Fig. 1). Those patients who also received primary arthroplasties in our hospital before revision arthroplasties were included in this study. We excluded patients undergoing primary arthroplasties with liver diseases, inflammatory and infectious arthritis, or incomplete data. Patients with PJI were diagnosed against the 2014 modified MusculoSkeletal Infection Society (MSIS) criteria in current study [15]. Patients without PJI were defined as cases undergoing single-stage revision for a diagnosis other than infection (loosening, wear, instability, malalignment, adverse local tissue reactions, or other aseptic causes).

### Clinical and serologic markers

Collected were demographic data, including gender, age, body mass index (BMI), smoking and drinking history, comorbidities, such as hypertension, cardiovascular, diabetes mellitus, kidney and thyroid diseases, indications for joint arthroplasty, pre-operative period of revision surgery, detailed information regarding PJI after primary arthroplasties and preoperative blood biomarkers, including total bilirubin [TB (µmol/L)], direct bilirubin [DB (µmol/L)], alanine aminotransferase [ALT (U/L)], aspartate aminotransferase [AST (U/L)], alkaline phosphatase [ALP (U/L)], glutamate-pyruvate transaminase [GGT (U/L)], creatinine (µmol/L), serum glucose (mmol/L), serum sodium (mmol/L), serum potassium (mmol/L), hemoglobin (g/L), RBC count (10^12^/L), WBC count (10^9^/L), blood platelet count (10^9^/L), CRP (mg/L), interleukin-6 [IL-6 (pg/ml)], ESR (mm/h), activated partial thromboplastin time [APTT (S)], fibrinogen (g/L) and D-dimer (µg/Ml). They were then analyzed and compared between the two groups. The serum TB and DB were detected by vanadate oxidation method on a fully automatic biochemical analyzer [16].

### Statistical analysis

All statistical analyses were performed by using SPSS Statistics 22.0 (IBM® Corporation, Armonk, NY, USA). Patients’ general data were presented by descriptive statistics. Categorical data were presented using numbers and quantitative data wereas presented as means plus standard deviation (SD) or range. For continuous and normally distributed data, the Student’s *t*-test was used. For ordinal or non-normally distributed data, the Mann–Whitney U test was applied. For categorical variables, a Pearson Chi-square or Fisher’s exact test was performed. A receiver operating characteristic (ROC) curve analysis was conducted to determine a possible cut-off point for preoperative bilirubin to distinguish between PJI and non-PJI patients. The area under the ROC curve (AUC) and Youden's index was assessed to better evaluate the diagnostic accuracy of preoperative bilirubin. An AUC < 0.5 was defined as less useful diagnostic test. Also, the odds ratio (OR) and corresponding 95% confidence intervals (CI) were calculated for diagnostic parameters.

## Results

### General information

We included 39 patients with PJI after primary THA (*n* = 13) or TKA (*n* = 26), compared with the contemporaneous 33 patients without PJI after primary THA (*n* = 17) or TKA (*n* = 16). The differences between two groups in age, gender, BMI, affected joints, smoking habit, alcohol habit and preoperative comorbidities were of no statistical significance, except the time from primary arthroplasty to revision surgery (Table 1). The time to revision in patients with and without PJI were 107.3 ± 28.25 weeks and 399.7 ± 49.46 weeks, respectively (*P* < 0.001).

**Table 1:** Comparisons of general information and comorbidities between cases and controls

	**Cases with PJI (*n* = 39)**	**Controls without PJI (*n* = 33)**	***P* value**
**Age**	58.59 ± 2.44	55.61 ± 2.60	0.406
**Gender**	21 F/18 M	16 F/17 M	0.650
**BMI (kg/m**^**2**^**)**	28.61 ± 1.84	25.67 ± 0.72	0.168
**Hip/Knee**	13 Hip / 26 Knee	17 Hip / 16 Knee	0.119
**Time to revision (W)**	107.3 ± 28.25	399.7 ± 49.46	** < 0.001**
**Smoking habit**	2	2	0.863
**Alcohol habit**	5	3	0.616
**Diabetes mellitus**	3	3	0.831
**Hypertension**	5	4	0.929
**Cardiovascular disease**	6	4	0.689
**Thyroid disease**	2	1	0.657
**ASA classification**	I 1 / II 34 / III 4	I 4 / II 27 / 2 III	0.233

*BMI* Body mass index, *ASA* American Society of Anesthesiologists

### Comparisons of preoperative serologic markers

All comparisons between patients with and without PJI regarding preoperative blood biomarkers are presented in Table 2. The only preoperative biomarkers which were significantly different between the PJI and non-PJI group were TB and DB. The preoperative TB in patients with and without PJI were 10.84 ± 0.61 μmol/L and 14.68 ± 0.75 μmol/L, respectively (*P* < 0.001). On the other hand, the preoperative DB was 3.07 ± 0.19 μmol/L and 4.70 ± 0.39 μmol/L in PJI group and no-PJI group (*P* < 0.001).

**Table 2:** Comparisons of preoperative blood biomarkers between cases and controls

	**Cases with PJI (*n* = 39)**	**Controls without PJI (*n* = 33)**	***P* value**
**Total bilirubin (μmol/L)**	10.84 ± 0.61	14.68 ± 0.75	** < 0.001**
**Direct bilirubin (μmol/L)**	3.07 ± 0.19	4.70 ± 0.39	** < 0.001**
**ALT (U/L)**	20.54 ± 1.97	20.53 ± 2.97	0.997
**AST (U/L)**	19.65 ± 1.90	19.27 ± 2.45	0.902
**ALP (U/L)**	78.67 ± 5.13	79.38 ± 8.82	0.943
**GGT (U/L)**	32.42 ± 3.36	37.07 ± 11.39	0.672
**Creatinine (μmol/L)**	65.69 ± 2.75	64.23 ± 2.04	0.682
**Serum glucose (mmol/L)**	5.23 ± 0.27	5.26 ± 0.24	0.931
**Serum sodium (mmol/L)**	141.10 ± 0.46	142.90 ± 0.55	0.156
**Serum potassium (mmol/L)**	3.84 ± 0.05	3.92 ± 0.07	0.361
**Hemoglobin (g/L)**	136.20 ± 1.72	140.70 ± 2.84	0.157
**RBC count (10**^**12**^**/L)**	4.47 ± 0.05	4.54 ± 0.09	0.501
**WBC count (10**^**9**^**/L)**	6.04 ± 0.27	6.18 ± 0.35	0.731
**Blood platelet count (10**^**9**^**/L)**	210.50 ± 8.85	207.50 ± 7.81	0.805
**CRP (mg/L)**	0.43 ± 0.07	0.49 ± 0.09	0.557
**IL-6 (pg/ml)**	6.59 ± 2.44	4.88 ± 1.08	0.712
**ESR (mm/h)**	10.27 ± 1.16	10.78 ± 1.34	0.773
**APTT (S)**	35.80 ± 0.79	35.47 ± 1.44	0.834
**Fibrinogen (g/L)**	3.24 ± 0.16	3.28 ± 0.18	0.861
**D-dimer (μg/Ml)**	0.71 ± 0.13	0.53 ± 0.11	0.543

*ALT* Alanine transaminase, *AST* Aspartate transaminase, *ALP* Alkaline phosphatase, *GGT* γ-glutamyl transferase, *RBC* Red blood cells, *WBC* White blood cells, *CRP* C-reactive protein, *IL* Interleukin, *ESR* Erythrocyte sedimentation rate, *APTT* Activated partial thromboplastin time

**Fig. 2:**
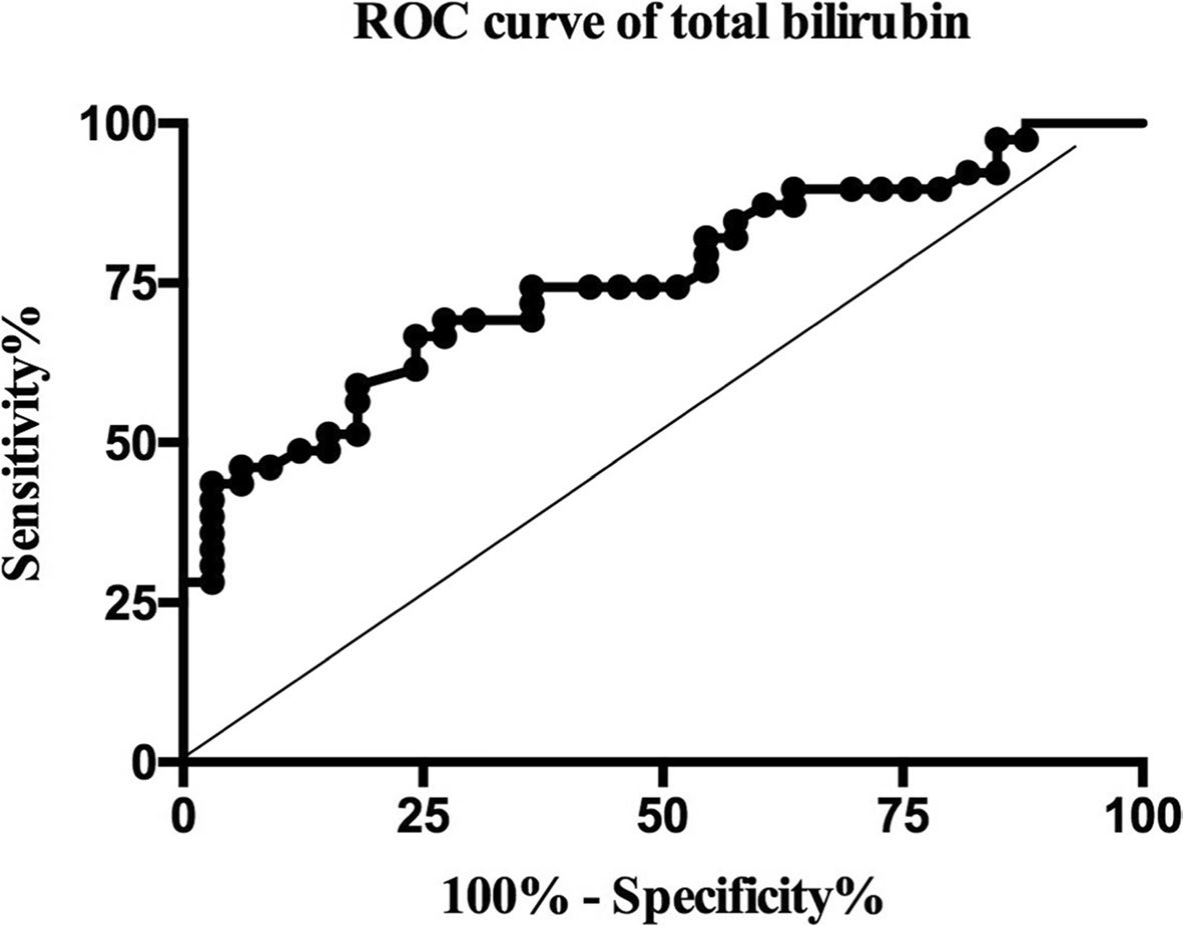
The ROC curve of total bilirubin to predict PJI. AUC = 0.755 (95%CI: 0.645–0.866, *P* < 0.001), Cutoff = 11.55 µmol/L, Sensitivity = 66.67%, Specificity = 75.76%, PPV = 76.47%, NPV = 65.79%, OR = 6.25 (95%CI: 2.21—17.65)

**Fig. 3:**
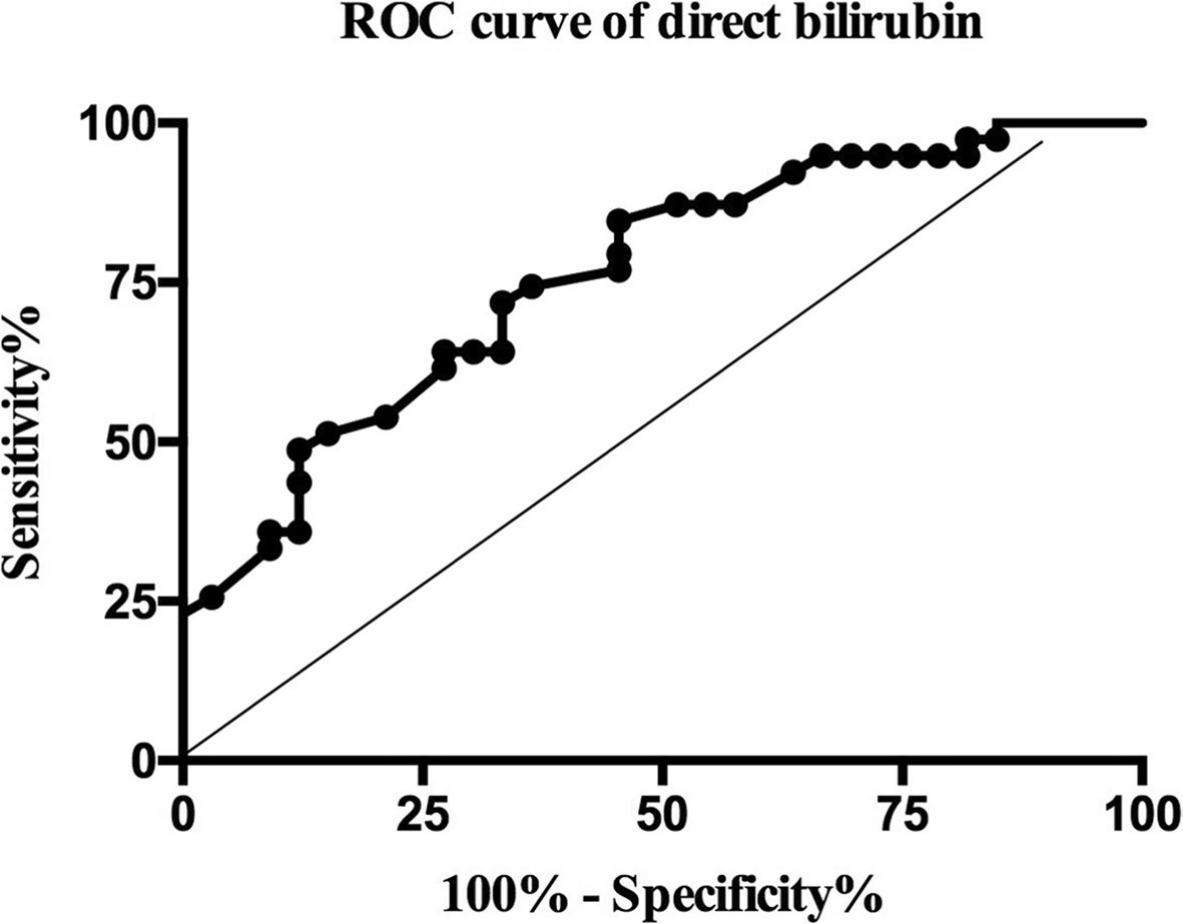
The ROC curve of direct bilirubin to predict PJI. AUC = 0.760 (95%CI: 0.651–0.870, *P* < 0.001), Cutoff = 4.00 µmol/L, Sensitivity = 84.62%, Specificity = 54.45%, PPV = 68.75%, NPV = 75%, OR = 6.60 (95%CI: 2.18—19.98)

The AUC for the preoperative TB levels to distinguish between PJI and non-PJI patients was 0.755 (95%CI: 0.645–0.866, *P* < 0.001; Fig. 2) and the cut-off value for a maximum of sensitivity and specificity was a preoperative TB level of 11.55 μmol/L (sensitivity: 66.67%, specificity: 75.76%, positive predictive value [PPV]: 76.47%, negative predictive value [NPV]: 65.79%, Youden's index: 0.424). The AUC for the preoperative DB levels to determine between PJI and non-PJI patients was 0.760 (95%CI: 0.651–0.870, *P* < 0.001; Fig. 3) and the cut-off value for a maximum of sensitivity and specificity was a preoperative TB level of 4.00 μmol/L (sensitivity: 84.62%, specificity: 54.45%, PPV: 68.75%, NPV: 75%, Youden's index: 0.391).

According to regression analysis of the case-control study, lower preoperative bilirubin levels (TB < 11.55 μmol/L or DB < 4.00 μmol/L) were significantly associated as a predictor for PJI (OR: 6.25, 95%CI: 2.21 to 17.65 or OR: 6.60, 95%CI: 2.18 to 19.98).

## Discussion

In this retrospective and case-control study, we evaluated preoperative blood biomarkers in 39 patients with PJI compared them with 33 patients without PJI after THA and TKA. The two groups were matched in terms of demographic and anatomical data (including age, gender, joint types), the potential risk factors (BMI, smoking habit and alcohol use) and other comorbidities. The main finding was that preoperative bilirubin levels were significantly lower in patients with PJI compared to controls without PJI after THA and TKA.

In 2018, Parvizi J et al [17] conducted an evidence-based study and validated new criteria for PJI diagnosis. The new criteria demonstrated a sensitivity of 97.7% and specificity of 99.5%. The PJI diagnostic process involved a multi-pronged and stepwise approach evaluating blood, synovial fluid, and tissue specimen tests. CRP and ESR are shown to be, as supported by strong evidence, a useful “ruling out” test [18, 19]. Synovial fluid tests such as leukocyte count and neutrophil percentage, leukocyte esterase, α-defensin, cultures, and next-generation sequencing for microorganisms can play an important diagnostic role [20–23]. If preoperative evaluation with serum and synovial fluid tests do not secure a diagnosis, the frozen section tissue histopathology may help establish PJI diagnosis [24].

Multiple risk factors were identified to be associated with PJI, including characteristics of the patient, surgical procedure and postoperative care [25]. A predictive model is a statistical equation that predicts an individual’s disease risk based on a combination of the values of multiple risk factors. Risk prediction models first originated in the area of cardiovascular disease prevention and have been widely used globally in clinical and public health practice [26]. Many predictive models for postoperative PJI have been developed [7]. Del Toro MD et al [27] developed and validated baseline, perioperative and at-discharge risk-scoring systems for PJI in patients undergoing arthroplasty. And they found that factors associated with PJI in the perioperative stage were THA, rheumatoid arthritis, obesity, National Nosocomial Infections Surveillance (NNIS) index > 2, significant wound bleeding and superficial surgical site infection. Tan TL et al [28] created a preoperative PJI risk calculator for assessing a patient's individual risk and found that factors such as prior surgical procedures and high-risk comorbidities should be considered when determining whether TJA is indicated and when counseling patients. However, available risk models to predict PJI have been developed using poor methodology and have several limitations, and they needs further validation using new data and its clinical effectiveness should be evaluated using a RCT design.

Kunutsor SK et al [7] proposed a predictive model that was mainly based on invasive data such as CRP, ESR and microbial etiology. In the current study, the preoperative CRP and ESR levels were 0.43 ± 0.07 and 10.27 ± 1.16 in PJI group and 0.49 ± 0.09 and 10.78 ± 1.34 in non-PJI group, respectively (*P* = 0.557 and *P* = 0.773). Many blood biomarkers are routinely assessed before joint arthroplasty, but it is unknown if many biomarkers proposed in the literature are possible of these biomarkers as possible predictors for postoperative PJI.

In this study, the most significant and main finding was that patients with mildly elevated preoperative bilirubin levels (TB ≥ 11.55 μmol/L or DB ≥ 4.00 μmol/L) were less prone to PJI, which was consistent with the result of Laky et al [14]. However, the research by Laky et al [14] has some limitations, including a small sample with only 18 PJI patients (8 shoulders and 10 knees), analysis of the total bilirubin alone and a definition of PJI that included the appearance of systemic or local signs for infection (*e.g.*, fever, and/or redness, swelling, heat, pain in the involved joint), combined with preoperative culture‐positive synovial fluid or tissues, sinus connected to the joint, intraoperative positive cultures, or positive frozen pathological section. The authors of the current study believe that mildly lower bilirubin, especially within normal range, may be used as a predictive factor for PJI after primary arthroplasties and, thus, should be further researched. For clinical application, mildly to moderately elevated bilirubin levels without signs of inflammation and increased liver biomarkers, bilirubin can be seen as protective factor for postoperative PJI.

Many epidemiological studies reported that higher bilirubin levels were related to reduced mortality and the protective role of bilirubin was explained by its anti-oxidative and anti-inflammatory capacities [13, 29]. The exact mechanisms are still unknown. Previous researches have explored the possible anti-inflammatory effect of bilirubin and found bilirubin might inhibit the production of pro-inflammatory cytokines (*e*.*g*. IL-6), which was in turn responsible for CRP production in the liver tissue [12, 13, 30]. Other studies on different pathologies such as cardiovascular diseases [29], metabolic syndrome and type 2 diabetes [30], cerebrovascular diseases [31], osteoporosis [32], and even on rheumatoid osteoarthritis [33] also showed a negative correlations with bilirubin concentrations. This has also been reported in studies evaluating the association between bilirubin and bacterial infections or associated models such as pathogen exposures, including endotoxin, although not all studies showed protective effects. The bilirubin levels were increased by stimulating heme oxygenase activity in animal model [34]. Therefore, it was speculated that increasing bilirubin levels within the normal range preoperatively in patients with arthroplasties has potential protective role because of its anti-oxidative and anti-inflammatory capacities, which would decrease the risk for PJI.

The current study has several limitations. First, this was a retrospective and single center study. Second, the study sample was 72 patients (39 patients with PJI and 33 patients without PJI). Thus, prospective multi-center study with larger sample will be needed to validate our finding. Third, underlying protective mechanisms of the mildly elevated bilirubin levels cannot be fully revealed by current study. We also know that the difference in bilirubin between PJI group and non-PJI group was small and that the mild reduction of bilirubin levels within normal range in the PJI group can only be identified as compared to the control group and not to the normal serum values. However, according to our research results, bilirubin seems a promising and easily available biomarker, which might be able to predict postoperative PJI.

## Conclusions

In summary, this retrospective study demonstrated that the levels of TB and DB before the primary joint replacement were lower in PJI patients than in non-PJI patients and bilirubin levels below a cut off at TB = 11.55 μmol/L or DB = 4.00 μmol/L could be considered as risk factors for postoperative PJI after primary THA and TKA.

## Data Availability

The datasets supporting the conclusions of this article are included within the article.

## Abbreviations

PJI: Periprosthetic joint infection
TB: Total bilirubin
DB: Direct bilirubin
TKA: Total knee arthroplasty
THA: Total hip arthroplasty
PPV: Positive predictive value
NPV: Negative predictive value
CRP: C-reactive protein
ESR: Erythrocyte sedimentation rate

## References

[1] Grammatico-Guillon L, Rusch E, Astagneau P (2015). Surveillance of prosthetic joint infections: international overview and new insights for hospital databases. J Hosp Infect.

[2] Delanois RE, Mistry JB, Gwam CU, Mohamed NS, Choksi US, Mont MA (2017). Current epidemiology of revision total knee arthroplasty in the United States. J Arthroplasty.

[3] Gwam CU, Mistry JB, Mohamed NS, Thomas M, Bigart KC, Mont MA (2017). Current epidemiology of revision total hip arthroplasty in the United States: national inpatient sample 2009 to 2013. J Arthroplasty.

[4] Huotari K, Peltola M, Jämsen E (2015). The incidence of late prosthetic joint infections: a registry-based study of 112,708 primary hip and knee replacements. Acta Orthop.

[5] Jämsen E, Varonen M, Huhtala H, Lehto MUK, Lumio J, Konttinen YT (2010). Incidence of prosthetic joint infections after primary knee arthroplasty. J Arthroplasty.

[6] Iannotti F, Prati P, Fidanza A, Iorio R, Ferretti A, Pèrez Prieto D, Kort N, Violante B, Pipino G, Schiavone Panni A, Hirschmann M, Mugnaini M, Francesco IP (2020). Prevention of periprosthetic joint infection (PJI): a clinical practice protocol in high-risk patients. Trop Med Infect Dis.

[7] Kunutsor SK, Whitehouse MR, Blom AW, Beswick AD (2017). Systematic review of risk prediction scores for surgical site infection or periprosthetic joint infection following joint arthroplasty. Epidemiol Infect.

[8] Kunutsor SK, Whitehouse MR, Blom AW, Beswick AD, INFORM Team (2016). Patient-related risk factors for periprosthetic joint infection after total joint arthroplasty: a systematic review and meta-analysis. PLoS One.

[9] Saleh A, George J, Faour M, Klika AK, Higuera CA (2018). Serum biomarkers in periprosthetic joint infections. Bone Joint Res.

[10] Kapitulnik J (2004). Bilirubin: an endogenous product of heme degradation with both cytotoxic and cytoprotective properties. Mol Pharmacol.

[11] Wagner K-H, Wallner M, Mölzer C, Gazzin S, Bulmer AC, Tiribelli C (2015). Looking to the horizon: the role of bilirubin in the development and prevention of age-related chronic diseases. Clin Sci.

[12] Arai T, Yoshikai Y, Kamiya J, Nagino M, Uesaka K, Yuasa N (2001). Bilirubin impairs bactericidal activity of neutrophils through an antioxidant mechanism in vitro. J Surg Res.

[13] Weinberger B, Archer FE, Kathiravan S, Hirsch DS, Kleinfeld AM, Vetrano AM (2013). Effects of bilirubin on neutrophil responses in newborn infants. Neonatology.

[14] Laky B, Alram I, Frank JK, Pauzenberger L, Anderl W, Wagner K-H (2020). Mildly decreased preoperative bilirubin levels are associated with infections after shoulder and knee arthroplasty. J Orthop Res.

[15] Parvizi J, Gehrke T, International Consensus Group on Periprosthetic Joint Infection (2014). Definition of periprosthetic joint infection. J Arthroplasty.

[16] Gu D, Wang Y, Ren B, Wang L, Zhang K, Yuan Y (2018). Comparison of three routine methods for the measurement of serum bilirubin in a China laboratory. Clin Lab.

[17] Parvizi J, Tan TL, Goswami K, Higuera C, Della Valle C, Chen AF (2018). The 2018 definition of periprosthetic hip and knee infection: an evidence-based and validated criteria. J Arthroplasty.

[18] Della Valle CJ, Sporer SM, Jacobs JJ, Berger RA, Rosenberg AG, Paprosky WG (2007). Preoperative testing for sepsis before revision total knee arthroplasty. J Arthroplasty.

[19] Greidanus NV, Masri BA, Garbuz DS, Wilson SD, McAlinden MG, Xu M (2007). Use of erythrocyte sedimentation rate and C-reactive protein level to diagnose infection before revision total knee arthroplasty. A prospective evaluation. J Bone Joint Surg Am.

[20] Ghanem E, Parvizi J, Burnett RSJ, Sharkey PF, Keshavarzi N, Aggarwal A (2008). Cell count and differential of aspirated fluid in the diagnosis of infection at the site of total knee arthroplasty. J Bone Joint Surg Am.

[21] Koh IJ, Han SB, In Y, Oh KJ, Lee DH, Kim TK (2017). The leukocyte esterase strip test has practical value for diagnosing periprosthetic joint infection after total knee arthroplasty: a multicenter study. J Arthroplasty.

[22] Trampuz A, Hanssen AD, Osmon DR, Mandrekar J, Steckelberg JM, Patel R (2004). Synovial fluid leukocyte count and differential for the diagnosis of prosthetic knee infection. Am J Med.

[23] Indelli PF, Ghirardelli S, Violante B, Amanatullah DF (2021). Next generation sequencing for pathogen detection in periprosthetic joint infections. EFORT Open Rev.

[24] Sigmund IK, Holinka J, Lang S, Stenicka S, Staats K, Hobusch G (2019). A comparative study of intraoperative frozen section and alpha defensin lateral flow test in the diagnosis of periprosthetic joint infection. Acta Orthop.

[25] Kong L, Cao J, Zhang Y, Ding W, Shen Y (2017). Risk factors for periprosthetic joint infection following primary total hip or knee arthroplasty: a meta-analysis. Int Wound J.

[26] Cook NR, Paynter NP, Eaton CB, Manson JE, Martin LW, Robinson JG (2012). Comparison of the Framingham and Reynolds Risk scores for global cardiovascular risk prediction in the multiethnic Women’s Health Initiative. Circulation.

[27] Del Toro MD, Peñas C, Conde-Albarracín A, Palomino J, Brun F, Sánchez S (2019). Development and validation of baseline, perioperative and at-discharge predictive models for postsurgical prosthetic joint infection. Clin Microbiol Infect.

[28] Tan TL, Maltenfort MG, Chen AF, Shahi A, Higuera CA, Siqueira M, Parvizi J (2018). Development and evaluation of a preoperative risk calculator for periprosthetic joint infection following total joint arthroplasty. J Bone Joint Surg Am.

[29] Horsfall LJ, Nazareth I, Petersen I (2012). Cardiovascular events as a function of serum bilirubin levels in a large, statin-treated cohort. Circulation.

[30] Nano J, Muka T, Cepeda M, Voortman T, Dhana K, Brahimaj A (2016). Association of circulating total bilirubin with the metabolic syndrome and type 2 diabetes: a systematic review and meta-analysis of observational evidence. Diabetes Metab.

[31] Higuchi S, Kabeya Y, Uchida J, Kato K, Tsukada N (2018). Low Bilirubin Levels indicate a high risk of cerebral deep white matter lesions in apparently healthy subjects. Sci Rep.

[32] Bian L-Q, Li R-Z, Zhang Z-Y, Jin Y-J, Kang H-W, Fang Z-Z (2013). Effects of total bilirubin on the prevalence of osteoporosis in postmenopausal women without potential liver disease. J Bone Miner Metab.

[33] Juping D, Yuan Y, Shiyong C, Jun L, Xiuxiu Z, Haijian Y (2017). Serum bilirubin and the risk of rheumatoid arthritis. J Clin Lab Anal.

[34] Wu BJ, Chen K, Barter PJ, Rye K-A (2012). Niacin inhibits vascular inflammation via the induction of heme oxygenase-1. Circulation.

[35] Kim DE, Lee Y, Kim M, Lee S, Jon S, Lee SH (2017). Bilirubin nanoparticles ameliorate allergic lung inflammation in a mouse model of asthma. Biomaterials.

[36] Wallner M, Bulmer AC, Mölzer C, Müllner E, Marculescu R, Doberer D (2013). Haem catabolism: a novel modulator of inflammation in Gilbert’s syndrome. Eur J Clin Invest.

