# Correction to: The mildly decreased preoperative bilirubin level is a risk factor for periprosthetic joint infection after total hip and knee arthroplasty

**DOI:** 10.1186/s42836-022-00122-x

**Published:** 2022-04-11

**Authors:** Jun Fu, Xiyue Chen, Ming Ni, Xiang Li, Libo Hao, Guoqiang Zhang, Jiying Chen

**Affiliations:** 1 Senior Department of Orthopedics, The Fourth Medical Center of Chinese PLA General Hospital, Beijing, China; 2 National Clinical Research Center for Orthopedics, Sports Medicine & Rehabilitation, Beijing, China; 3 Department of Orthopedics, The First Medical Center of Chinese PLA General Hospital, Beijing, China; 4 Department of Orthopaedics, Sanya People’s Hospital, Sanya, 572000 China

**Correction to: Arthroplasty 3, 1-8 (2021)**

**https://doi.org/10.1186/s42836-021-00096-2**

Following publication of the original article [1], some information was missing in the Competing interests section.

The updated Competing interests is given below:

**Competing interests**

Guoqiang Zhang and Jiying Chen are members of the Editorial Board of Arthroplasty and other authors declare that they have no competing interests. All authors were not involved in the journal’s review of or decisions related to this manuscript.

The original article [1] has been corrected.
